# GLP-1 Receptor Agonist Improves Mitochondrial Energy Status and Attenuates Nephrotoxicity In Vivo and In Vitro

**DOI:** 10.3390/metabo13111121

**Published:** 2023-11-01

**Authors:** Linxi Wang, Zhou Chen, Xiaoying Liu, Lijing Wang, Yu Zhou, Jingze Huang, Zhiqing Liu, Donghai Lin, Libin Liu

**Affiliations:** 1 Department of Endocrinology and Metabolism, Fujian Institute of Endocrinology, Fujian Medical University Union Hospital, Fuzhou 350001, China; wanglinxi@fjmu.edu.cn (L.W.); liuxiaoyin@fjmu.edu.cn (X.L.); wanglijing@fjmu.edu.cn (L.W.); cfjsczy@fjmu.edu.cn (Y.Z.); huangjingze@fjmu.edu.cn (J.H.); 2 Department of Pharmacology, College of Pharmacy, Fujian Medical University, Fuzhou 350001, China; chenzhou@fjmu.edu.cn; 3 Key Laboratory for Chemical Biology of Fujian Province, College of Chemistry and Chemical Engineering, Xiamen University, Xiamen 361005, China; 20520151152315@stu.xmu.edu.cn (Z.L.); dhlin@xmu.edu.cn (D.L.)

**Keywords:** GLP-1, kidney injury, metabolism, mitochondrial, energy metabolism

## Abstract

High-sugar and high-fat diets cause significant harm to health, especially via metabolic diseases. In this study, the protective effects of the antidiabetic drug exenatide (synthetic exendin-4), a glucagon-like peptide 1 (GLP-1) receptor agonist, on high-fat and high-glucose (HFHG)-induced renal injuries were investigated in vivo and in vitro. In vivo and in vitro renal injury models were established. Metabolomic analysis based on ^1^H-nuclear magnetic resonance was performed to examine whether exenatide treatment exerts a protective effect against kidney injury in diabetic rats and to explore its potential molecular mechanism. In vivo, 8 weeks of exenatide treatment resulted in the regulation of most metabolites in the diabetes mellitus group. In vitro results showed that exendin-4 restored the mitochondrial functions of mesangial cells, which were perturbed by HFHG. The effects of exendin-4 included the improved antioxidant capacity of mesangial cells, increased the Bcl-2/Bax ratio, and reduced protein expression of cyt-c and caspase-3 activation. In addition, exendin-4 restored mesangial cell energy metabolism by increasing succinate dehydrogenase and phosphofructokinase activities and glucose consumption while inhibiting pyruvate dehydrogenase E1 activity. In conclusion, GLP-1 agonists improve renal injury in diabetic rats by ameliorating metabolic disorders. This mechanism could be partially related to mitochondrial functions and energy metabolism.

## 1. Introduction

The total estimated prevalence of diagnosed and undiagnosed diabetes is 10.9% in the adult Chinese population [1]. Because high-fat and high-sugar diets are becoming common, the prevalence of diabetes is increasing every year. Diabetic kidney disease (DKD) is one of the most serious microvascular complications of diabetes mellitus (DM). DKD often coexists with albuminuria or proteinuria and has become the leading cause of end-stage renal disease worldwide [2]. Numerous factors contribute to the development of DKD, such as oxidative stress, the accumulation of advanced glycation end products, chronic inflammation [3,4], metabolic disorders [5], and podocyte injury [6]. Several metabolic disorders, such as chronic hyperglycemia and dyslipidemia, are not only major symptoms of DKD, but are also considered important initiators of its pathogenesis [7]. In the present study, the protective effects of the antidiabetic drug exenatide (synthetic exendin-4), a glucagon-like peptide 1 (GLP-1) receptor agonist, on high-fat and high-glucose (HFHG)-induced renal injuries were investigated.

Among the organs, the kidney has the second highest energy consumption rate. The kidneys consume a large amount of energy to regulate blood pressure and body fluids by excreting waste products and toxins [8]. Therefore, it is important to balance energy supply and demand; otherwise, kidney damage may occur [9]. Mitochondrial dysfunction is correlated with abnormal renal repair in chronic kidney disease. Various quality control mechanisms, such as antioxidant defense, protein quality control, mitochondrial dynamics, mitochondrial autophagy, and mitochondrial biosynthesis, enable the mitochondria to sustain homeostasis under both physiological and pathological conditions [10]. It is imperative to maintain these quality control mechanisms to ensure proper mitochondrial function. Disrupting these mechanisms can result in mitochondrial harm and impairment, leading to cell mortality, tissue damage, and organ dysfunction. A plethora of evidence implies that disturbing the mechanisms that initiate mitochondrial quality control has a substantial role in the pathogenesis of DKD [11]. Therapeutic approaches aimed at maintaining mitochondrial quality control mechanisms are currently being developed and have encouraging potential for treating and preventing DKD and boosting renal restoration. Nevertheless, the unclear mechanisms and potential adverse effects make it difficult to translate these findings into clinical practice.

GLP-1 is an incretin, which is a gut hormone secreted from the L cells of the intestine in response to food intake. Increasing studies have found that GLP-1 has immune-regulatory, anti-inflammatory, and antihypertensive effects [12]. Exenatide, a GLP-1 receptor agonist, is beneficial for the prevention of diabetic complications [13]. Increasing evidence suggests that GLP-1 also has beneficial effects on renal disease [14]. One clinical trial showed that the rates of new or worsening nephropathy were lower in the GLP-1 receptor agonist semaglutide group than in the placebo group [15]. Further evidence shows that GLP-1 agonists can exert an inhibitory effect on mesangial cell proliferation and fibronectin (FN) secretion through AMPK signaling [16], which is an important pathway for energy metabolism. GLP-1 receptor agonists are thus likely to effectively alleviate diabetic renal damage through their pleiotropic effects on metabolic regulation [17].

A metabolomic analysis can provide important global insight into stimulus-induced metabolic shifts in different physiological or pathological states before histopathological changes are detected, which could contribute to the identification of potential targets for antidiabetic drugs [18]. The discovery and study of many metabolites not only provide a better understanding of the pathophysiology of DKD but also raise hope for its prognosis or early diagnosis. In the current study, metabolomics was considered an effective tool to identify DKD metabolic features that are crucial for its prevention and treatment [19].

In the present study, we established in vivo and in vitro renal injury models. A ^1^H-nuclear magnetic resonance (NMR)-based metabolomic analysis was performed to examine whether exenatide treatment exerts a protective effect on kidney injury in diabetic rats and to explore its potential molecular mechanism.

## 2. Materials and Methods

### 2.1. Animals and Treatments

Male Sprague–Dawley rats (180 ± 20 g) were purchased from the Fujian Provincial Centre for Disease Control and Prevention. Animals were housed in a well-ventilated, temperature-controlled environment at 22 ± 2 °C with a 12 h light–dark cycle. All animal experimental protocols were approved by the Institutional Animal Ethics Committee of Fujian Medical University and conformed with the guidelines of the NIH Guide for the Care and Use of Laboratory Animals.

**Figure 1:**
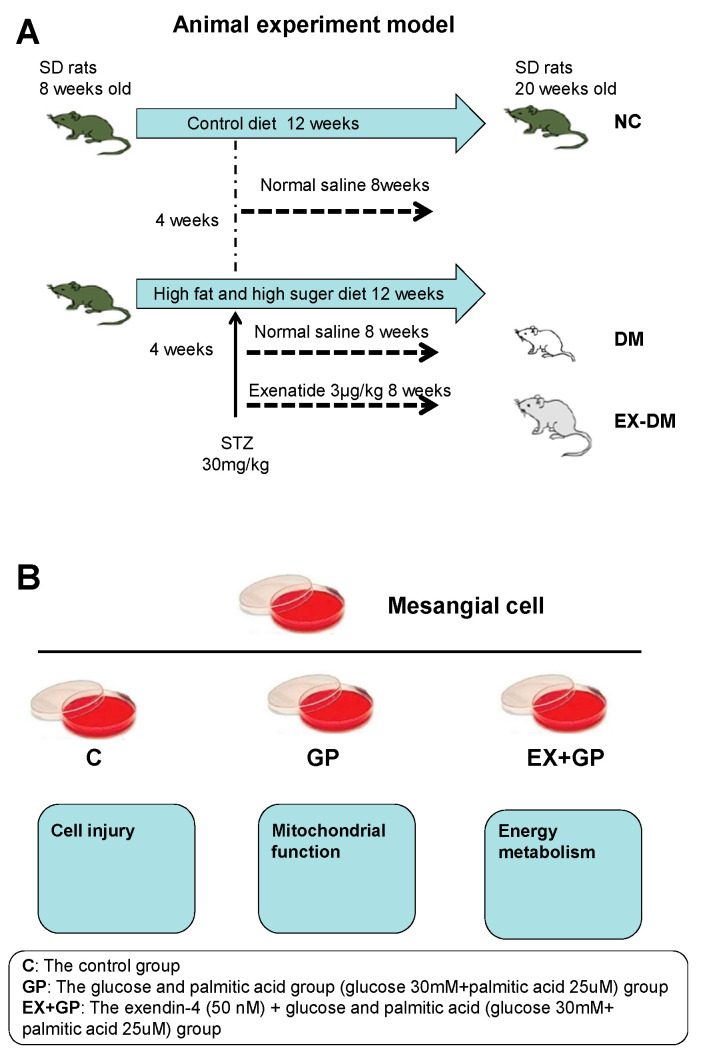
Experimental design. (**A**) Establishment and grouping of animal models. (**B**) Establishment and grouping of cell models. NC: control; DM: diabetes mellitus (DM)-model rats; ex-DM: exenatide-treated DM-model rats; C: control; GP: high-fat high-glucose (HFHG); EX-GP: exendin-4 + HFHG.

The rats were randomly assigned to receive either the standard chow diet in the normal control (NC) group (*n* = 6) or an HFHG diet (containing 67.5% basic feed, 20% sugar, 10% lard, and 2.5% egg yolk; *n* = 12) for 4 weeks. Rats fed the HFHG diet were intraperitoneally injected with streptozotocin (STZ; 30 mg/kg, diluted in 0.1 mol/L citrate buffer, pH 4.4; Sigma-Aldrich, St. Louis, MO, USA). Three days later, blood from fasted rats was collected, and glucose levels were measured. Rats with fasting blood glucose levels ≥16.7 mmol/L were classified as having DM and were fed an HFHG diet in subsequent studies. The rats with DM were randomly divided into two groups (*n* = 6 per group) as follows: DM, in which the rats were treated with normal saline; and ex-DM, in which the rats were treated with exenatide (3 μg/kg, twice daily; Baxter Pharmaceutical Solution LLC, Deerfeld, IN, USA) via subcutaneous administration for 8 weeks. Meanwhile, the NC group received normal saline treatment (Figure 1A).

### 2.2. Cell Culture and Treatments

Mouse glomerular mesangial cells (SV40 MES 13) were purchased from the Typical Cell Culture Collection Committee of the Chinese Academy of Sciences Library. The cells were maintained in Dulbecco’s modified Eagle medium (DMEM; which contains 5.5 mmol/L glucose) supplemented with 5% fetal bovine serum (FBS), 100 units/mL penicillin, and 100 μg/mL streptomycin and cultured at 37 °C in humidified air with 5% CO_2_. Upon reaching 75% confluency, the cells were passaged once every 3 d. The cells were divided into the following groups 24 h after synchronization in a serum-free culture medium: (1) control group (5.5 mM glucose); (2) GP group (30.0 mM glucose + 25 µM palmitic acid); (3) EX + GP group (50 nM exendin-4 + 30.0 mM glucose + 25 µM palmitic acid); (4) M group (5.5 mmol/L glucose + 24.5 mmol/L mannitol); and (5) EX group (50 nM exendin-4 alone). SV40 MES 13 cells were preincubated with 50 nM exendin-4 for 1 h before exposure to GP for 24 h (Figure 1B).

### 2.3. Biological Parameters

At the end of the experiment, urine samples were collected by placing the animals in metabolic cages with water for 24 h. Urinary albumin (UA) levels were measured using an IMMAGE 800 Specific Protein Analysis Kit (Beckman Coulter Inc., Bria, CA, USA). Fasting blood glucose levels were measured weekly using an Optium Xceed blood glucose meter (Abbott Diabetes Care Inc., Chicago, IL, USA). Blood samples were collected from the caudal veins, and plasma glycated hemoglobin (HbA1c) levels were measured using a rat HbA1c enzyme-linked immunosorbent assay (ELISA) kit (Westang Bio-Tech Inc., Shanghai, China). The serum was prepared by centrifuging the blood samples at 3000 rpm for 15 min. Serum levels of triglycerides, total cholesterol, high-density lipoprotein (HDL), low-density lipoprotein (LDL), blood urea nitrogen (BUN), and creatinine (CREA) were measured using the corresponding assay kits and an automatic biochemical analyzer (LX20; Beckman Coulter Inc.). Blood samples were collected via abdominal aortic puncture under anesthesia.

### 2.4. Preparation of Aqueous Kidney Extracts and Acquisition of ^1^H NMR Spectra

Prior to NMR analysis, the frozen kidney tissue was weighed, ground in a mortar, and prepared as described previously [20].

### 2.5. Cell Proliferation Assay

Mesenchymal cells were seeded at a density of 1 × 10^4^ cells/well in DMEM containing 5% FBS. The cell growth rate was measured using Cell Counting Kit-8 (Beyotime Biotech Co., Ltd., Haimen, China) according to the manufacturer’s instructions. Absorbance was measured at 450 nm using an ELx800 microplate reader.

### 2.6. Hoechst 33342 Staining

Apoptotic cells were stained with Hoechst 3334 (Beyotime Biotech), and the apoptotic rate was calculated by counting 100 cells at three independent time points according to the manufacturer’s instructions.

### 2.7. ELISA

Cell culture supernatants from the different treatment groups were collected and centrifuged at 2000× *g* for 20 min. The supernatants were screened using a commercial ELISA kit (Shanghai EnzymeLink Biotechnology Co., Ltd., Shanghai, China) according to the manufacturer’s instructions, and the absorbance was finally measured at 450 nm using an ELx800 microplate reader. Pyruvate dehydrogenase El activity, phosphofructokinase activity, glucose, lactic acid, and succinate hydrogenase activity were detected using ELISA.

### 2.8. Western Blotting

The proteins isolated from cells were separated on sodium dodecyl sulfate-polyacrylamide gels, transferred onto polyvinylidene fluoride membranes, and incubated overnight at 4 °C with primary antibodies against Bcl-2 (1:500), Bax (1:1000), cyt-c (1:1000), active caspase-3 (1:500), total-caspase-3 (1:500), or β-actin (1:1000). Band densities of target proteins were analyzed using Image Pro Plus 6.0 software (Media Cybernetics; MD, Rockville, MD, USA).

### 2.9. Statistical Analysis

Data are presented as the mean ± standard deviation. The Kolmogorov–Smirnov algorithm was used to evaluate the normality of each metabolite concentration. Comparisons among groups were conducted using one-way analysis of variance with Tukey’s post hoc test. Statistical analysis was performed using GraphPad Prism software (version 5; Graphpad Software Inc., San Diego, CA, USA). Statistical significance was set at *p* < 0.05.

## 3. Results

### 3.1. Effects of Exenatide on Plasma HbA1c Levels, Serum Fasting Blood Glucose, and Lipid Profiles in Rats with DM

**Figure 2:**
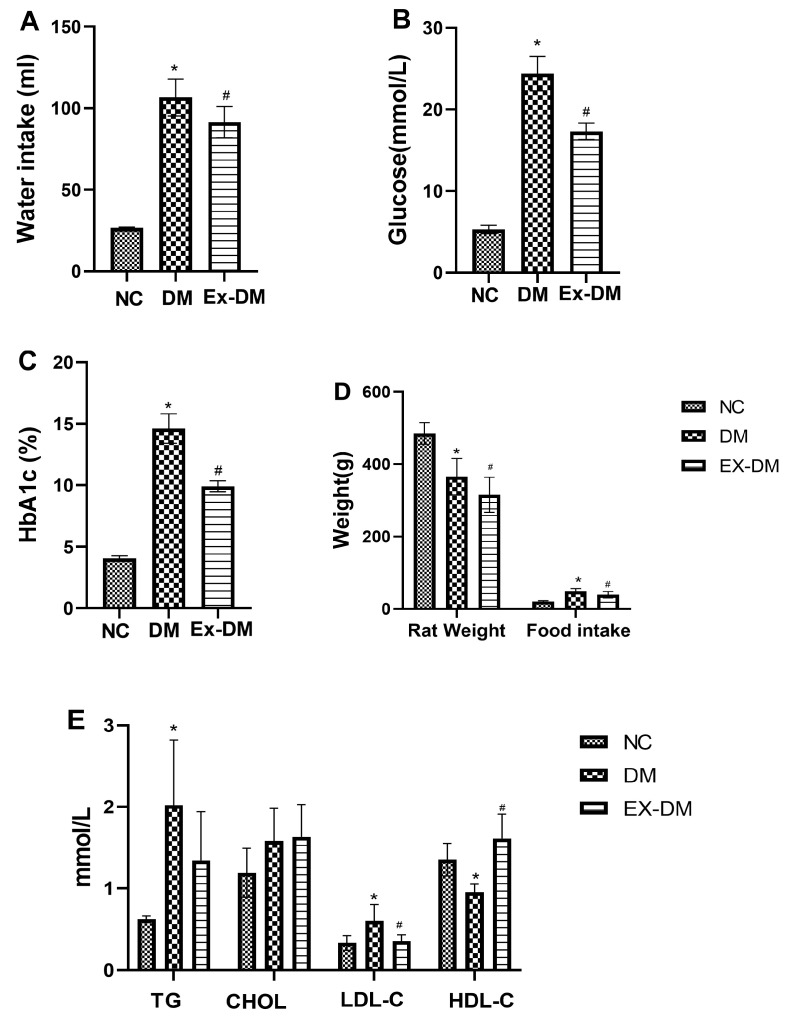
Effect of exenatide on general and clinical parameters in rats. Water intake (**A**), glucose (**B**), HbA1c (**C**), weight (**D**), the serum lipid level (**E**) of mice in each group. Data are expressed as the mean ± S.D. (*n* = 6/group). Statistical significance: * *p* < 0.05 vs. NC, # *p* < 0.05 vs. DM. NC: control; DM: diabetes mellitus (DM)-model rat group; EX-DM: exenatide-treated DM-model rat group; TG, triglycerides; CHOL, cholesterol; LDL, low-density lipoprotein; HDL, high-density lipoprotein.

During the entire period of the experiment, the DM group showed increased food and water intake and weight loss, whereas the ex-DM group showed significantly reduced food and water intake and exhibited weight gain. The blood glucose and HbA1c levels in the ex-DM group were lower than those in the DM group (Figure 2). Dyslipidemia is defined as an altered metabolism in DM. We examined the effects of exenatide treatment on serum lipid profiles in all DM-model rats and found reduced LDL-C levels and increased HDL levels in the ex-DM group (*p* < 0.05) compared with those in the DM group.

### 3.2. Effects of Exenatide on Kidney Functions in DM-Model Rats

As illustrated in Table 1, the renal index in the DM group was higher than that in the NC and ex-DM groups. Moreover, as a characteristic feature of the early stage of diabetic nephropathy, the 24 h UA level in the DM group was significantly higher than that in the NC group (*p* < 0.05). Of note, the 24 h UA level was lower in the ex-DM group than in the DM group due to exenatide treatment at 8 weeks. However, BUN and CREA levels did not significantly differ among the three groups.

### 3.3. Exenatide Ameliorates Renal Metabolic Disorders in DM-Model Rats

**Figure 3:**
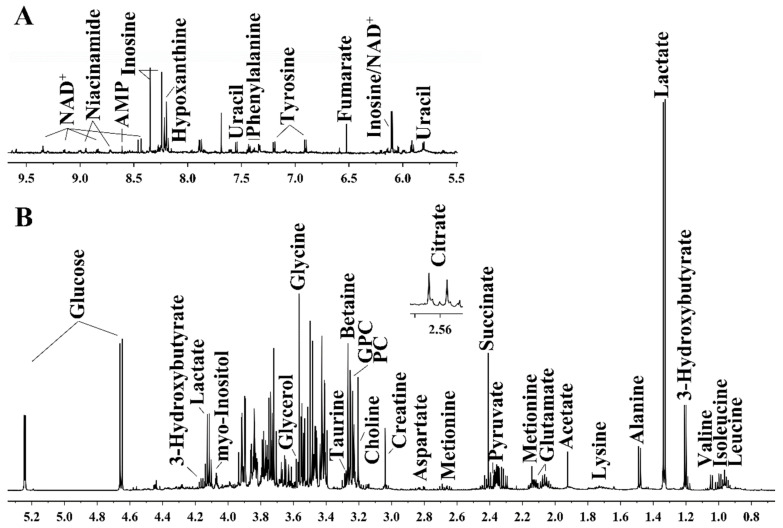
Representative 1D ^1^H-NMR (nuclear magnetic resonance) spectra (600 MHz) of rat kidney extracts. (**A**) Region of 5.5 to 9.53 ppm; this region has been magnified 15 times compared to region B. (**B**) Region of 0.7 to 5.3 ppm. The inset shows an additional signal detected in the spectra obtained for the other sample groups.

**Figure 4:**
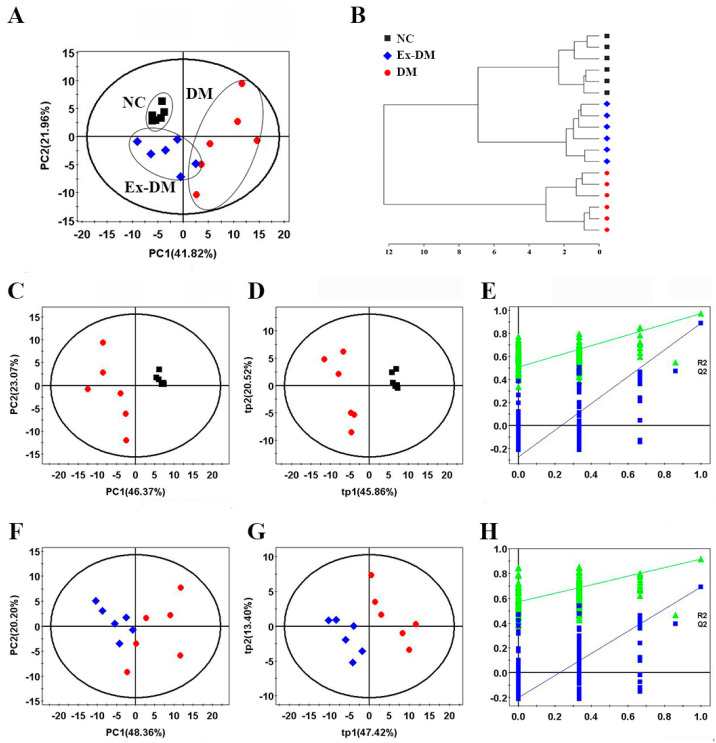
Pattern recognition analysis of the three groups. (**A**) PCA score plot of the three groups. (**B**) Hierarchical cluster analysis results. (**C**) PCA score plot, (**D**) PLS-DA score plot (R^2^X = 0.664, R^2^Y = 0.97, Q^2^ = 0.889), and (**E**) PLS-DA cross-validation plot from DM and NC groups. (**F**) PCA score plot, (**G**) PLS-DA score plot (R^2^X = 0.603, R^2^Y = 0.915, Q^2^ = 0.692), and (**H**) PLS-DA cross-validation plot from the ex−DM and DM groups. The ellipses indicate the 95% confidence limit. Cross-validation plots were generated from permutation tests (*n* = 200) using the first two components (tp1 and tp2). NC, control; DM, diabetes mellitus (DM)−model rats; Ex−DM, exenatide-treated DM-model rats.

**Figure 5:**
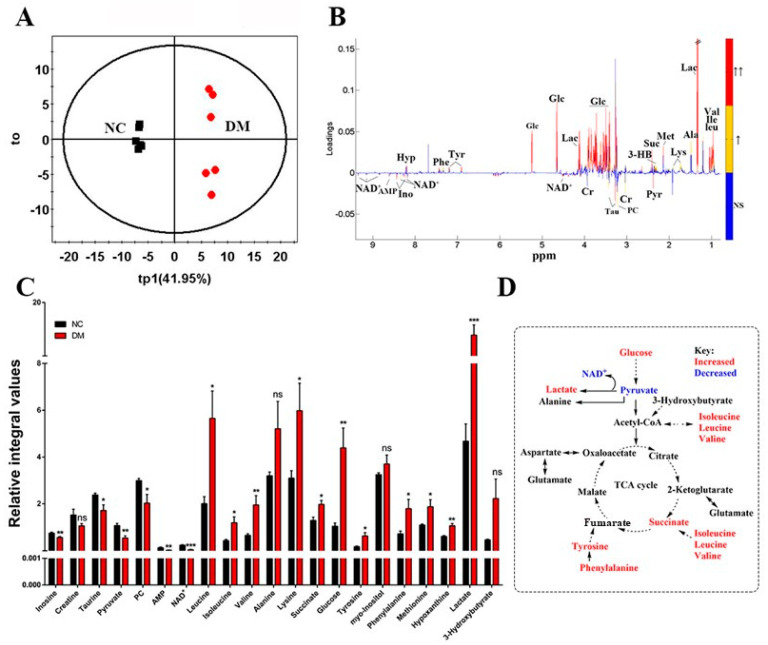
Identification of characteristic metabolites significantly responsible for discriminating between the metabolic profiles of the DM and NC groups. (**A**) OPLS−DA score plot. (**B**) OPLS−DA correlation coefficient-coded loading plot identifying metabolites that significantly contributed to the discrimination of metabolic profiles. Red, orange, and blue indicate that the variables are very significant (|r| ≥ 0.708 and VIP ≥ 1), significant (B: 0.576 ≤ |r| < 0.708 and VIP ≥ 1), and insignificant, respectively. (**C**) Quantitative analysis of the characteristic metabolites between the two groups (Student’s *t*-test was used: * *p* < 0.05, ** *p* < 0.01, *** *p* < 0.001 with respect to the NC group, ns means no statistical significance). (**D**) Metabolic pathway schematic highlighting the metabolites that were differentially regulated by DM. Abbreviations: Val, valine; Ile, isoleucine; Leu, leucine; Lac, lactate; Ala, alanine; Lys, lysine; Met, methionine; Suc, succinate; 3-HB, 3-hydroxybutyrate; Glc, glucose; Tyr, tyrosine; Phe, phenylalanine; Hyp, hypoxanthine; Pyr, pyruvate; Cr, creatine; PC, O−phosphocholine; TAU, taurine; Ino, inosine; NAD^+^, nicotinamide adenine dinucleotide; AMP, adenosine monophosphate. NC, control; DM, diabetic mellitus (DM)−model rat group.

**Figure 6:**
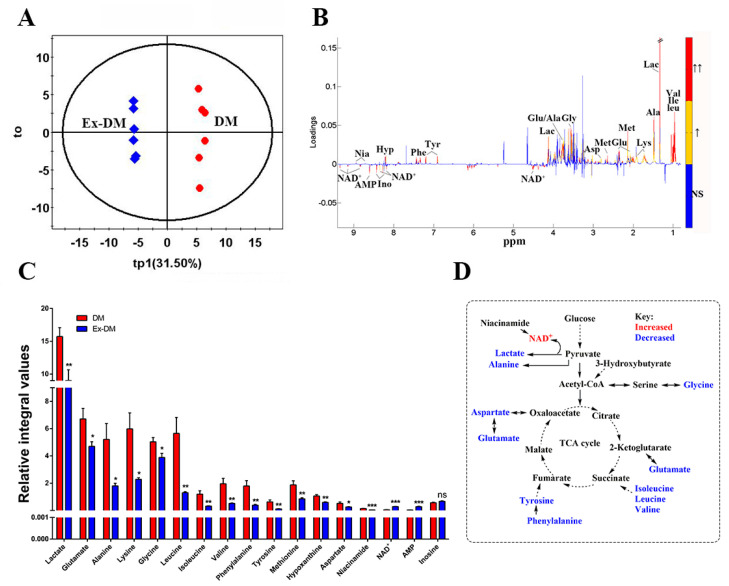
Identification of characteristic metabolites significantly responsible for discriminating between the metabolic profiles of the ex−DM and DM groups. (**A**) OPLS−DA score plot. (**B**) OPLS−DA correlation coefficient-coded loading plot identifying metabolites that significantly contributed to the discrimination of metabolic profiles. Red, orange, and blue indicate that the variables are very significant (|r| ≥ 0.708 and VIP ≥ 1), significant (B: 0.576 ≤ |r| < 0.708 and VIP ≥ 1), and insignificant, respectively. (**C**) Quantitative analysis of the characteristic metabolites between the two groups (Student’s *t*-test was used: * *p* < 0.05, ** *p* < 0.01, *** *p* < 0.001 with respect to the NC group, ns means no statistical significance). (**D**) Metabolic pathway schematic highlighting the metabolites that were differentially regulated in the ex-DM group. Abbreviations: Val, valine; Ile, isoleucine; Leu, leucine; Lac, lactate; Ala, alanine; Lys, lysine; Met, methionine; Glu, glutamate; Asp, aspartate; Gly, glycine; Tyr, tyrosine; Phe, phenylalanine; Hyp, hypoxanthine; Nia, niacinamide; Ino, inosine; NAD^+^, nicotinamide adenine dinucleotide; AMP, adenosine monophosphate. DM: diabetic mellitus (DM)−model rat group, Ex−DM: exenatide−treated DM−model rat group.

The identified metabolites based on the ^1^H NMR spectra of the aqueous extracts from the rat kidneys are shown in Figure 3 and Table 2. Orthogonal partial least-squares discriminant analysis score plots showed clear clustering among the three groups (Figure 4). In the aqueous extract, the DM group showed higher levels of glucose, lactate, valine, isoleucine, leucine, lysine, methionine, succinate, tyrosine, and phenylalanine, and lower levels of pyruvate, nicotinamide adenine dinucleotide (NAD^+^), AMP, taurine (TAU), phosphocholine (PC), and inosine than those in the control group (Figure 5). Meanwhile, the ex-DM group exhibited improvements in almost all of these abnormal amino acid metabolites, as well as increased NAD^+^ and AMP levels and decreased lactate and glucose levels compared with those in the DM group (Figure 6). When we compared the metabolite profiles of the ex-DM group to those of the NC group, we found that the metabolites were similar, suggesting that exenatide might exert a protective effect against DM.

### 3.4. Exendin-4 Inhibits HFHG-Induced Injury in Mesangial Cells

To validate the effects of the GLP-1 agonist exendin-4 on mitochondrial function, we conducted an in vitro analysis using mesangial cells. There was no statistical difference among the M, EX, and control groups, and the possibility of cell injury caused by hyperosmolar and exendin-4 was excluded. We used the control, GP, and EXGP groups for subsequent research.

**Figure 7:**
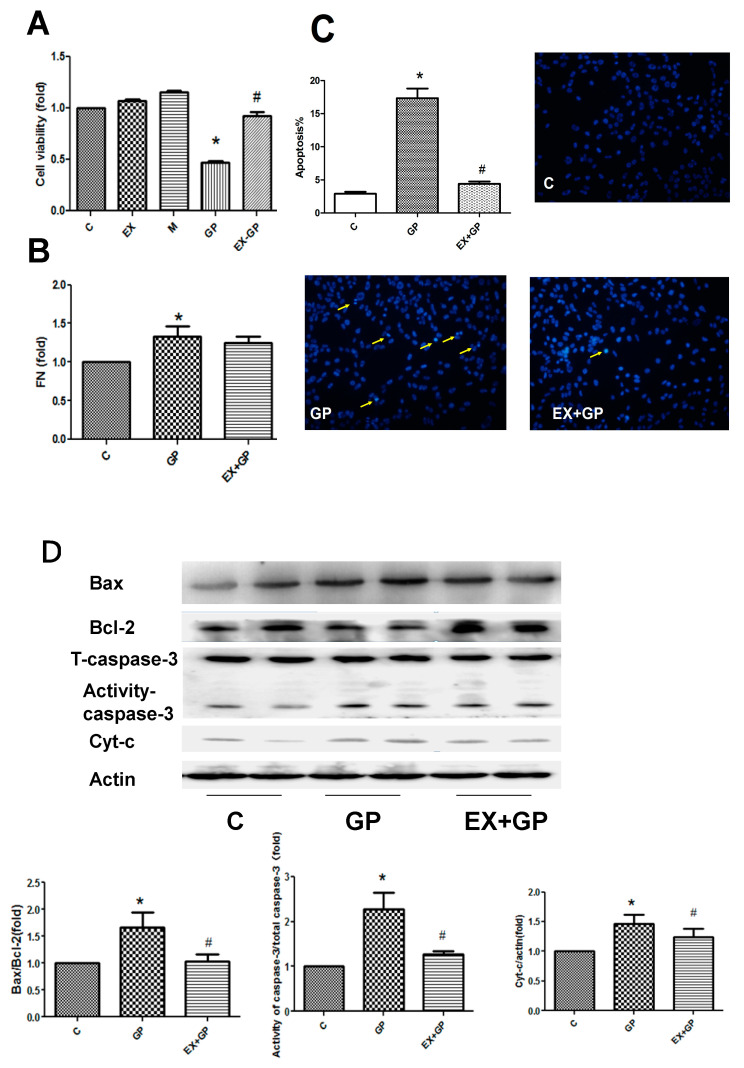
Exendin-4 inhibits high-fat high-glucose (HFHG)-induced injury in mesangial cells. (**A**) Cell viability was assessed using the CCK-8 assay. (**B**) FN levels were measured using an ELISA kit. (**C**) The rate of apoptosis was determined using Hoechst staining. The yellow arrows show apoptotic cells. (**D**) The apoptotic pathways were assessed using Western blotting. Cells containing bright-blue particles (apoptotic cells: yellow arrows) were visualized under a microscope, and the data are expressed as the mean ± S.D. (*n* = 3/group). Statistical significance: * *p* < 0.05 vs. C, # *p* < 0.05, vs. GP. C: control, EX: exendin-4 alone, M: mannitol, GP: HFHG, EX+GP: exendin-4 + HFHG.

We first examined the effect of exendin-4 on mesangial cell injury. HFHG-treated mesangial cells showed decreased cell survival rates, increased apoptosis rates, and increased FN expression. These effects were reversed by treatment with exendin-4, implying that exendin-4 can alleviate HFHG-induced damage to mesangial cells (Figure 7A,B). We then examined the apoptotic pathways. The results showed that HFHG increased the Bax/Bcl-2 ratio and the concentration of active caspase-3 and upregulated cyt-c expression, leading to an increase in apoptosis; these effects were reversed by exendin-4 pretreatment (Figure 7D).

### 3.5. Effect of Exendin-4 on Mitochondrial Function in Mesangial Cells

**Figure 8:**
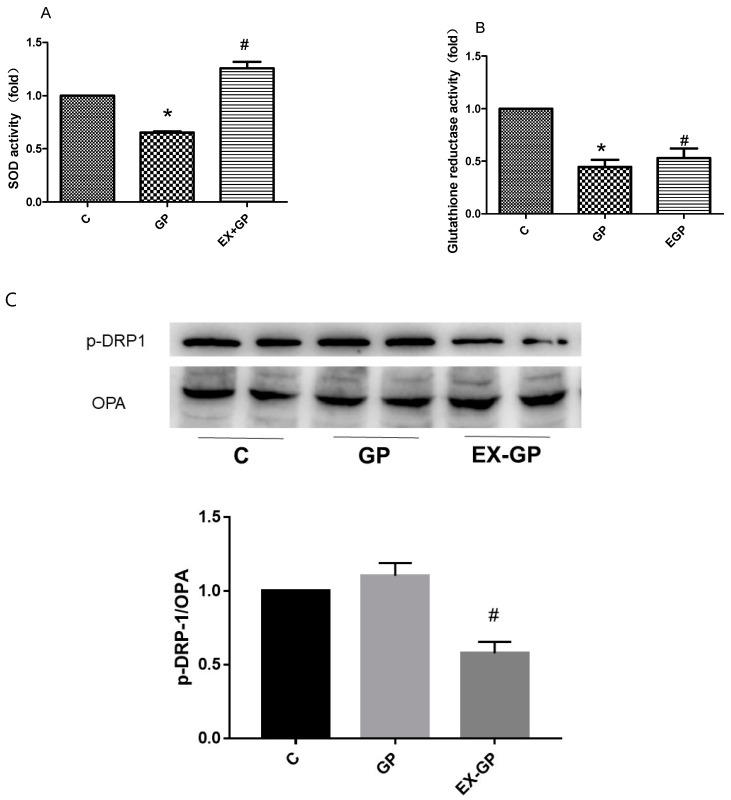
Effect of exendin-4 on the antioxidant ability of mesangial cells. (**A**) SOD activity was measured using a Superoxide Assay Kit, and (**B**) GR activity was measured using the Glutathione Reductase Assay Kit. (**C**) Western blotting detection of mitochondrial kinetic protein (p-DRP/OPA) expression. Data are expressed as the mean ± S.D. (*n* = 3/group). Statistical significance: * *p* < 0.05 vs. C, # *p* < 0.05, vs. GP. C: control, GP: high-fat high-glucose (HFHG) group, EX-GP: exendin-4 + HFHG.

As mitochondria are an important site of the TCA cycle, it is important to study whether their function changes in an environment with HFHG. We observed the effect of HFHG on the antioxidant capacity of mesangial cells. We found that superoxide dismutase and glutathione reductase activities were reduced upon HFHG treatment, and the antioxidant capacity of mesangial cells was restored after exendin-4 pretreatment. These results suggest that exendin-4 plays a role in protecting mesangial cells by improving mitochondrial function (Figure 8A,B). Mitochondrial function is closely related to mitochondrial dynamics. We measured two proteins associated with mitochondrial dynamics, and the results suggested that exendin-4 treatment decreased the ratio of p-DRP/OPA, increased mitochondrial fusion, and improved mitochondrial homeostasis; these results suggest that exendin-4 might protect mesangial cells by improving mitochondrial function (Figure 8C).

### 3.6. Exendin-4 Improves Glucose Metabolism and the TCA Cycle in Mesangial Cells

**Figure 9:**
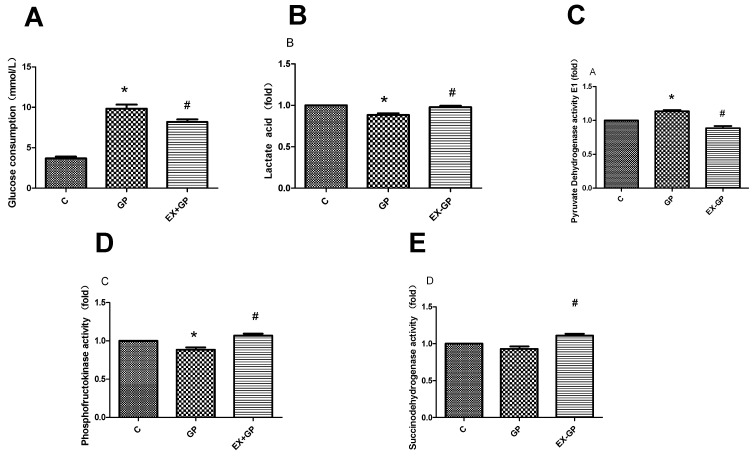

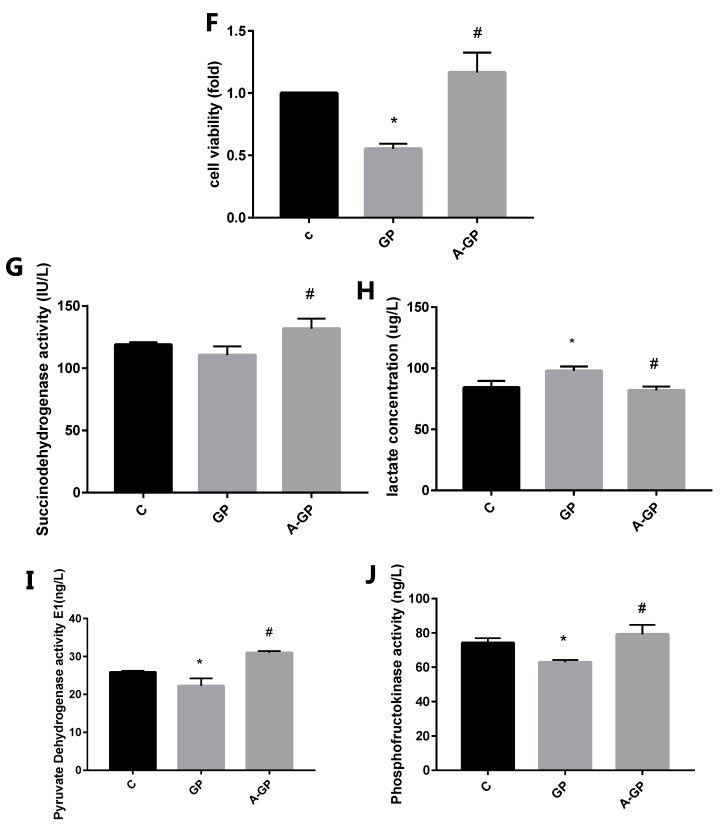
Effect of exendin-4 on energy metabolism in mesangial cells. (**A**) Glucose consumption, (**B**) lactic acid concentration, (**C**) pyruvate dehydrogenase E1 activity, (**D**) phosphofructokinase activity and (**E**) succinodehydrogenase activity in each group, were measured using an ELISA kit. (**F**) Cell viability, (**G**) succinodehydrogenase activity, (**H**) lactic acid concentration, (**I**) pyruvate dehydrogenase E1 activity, and (**J**) phosphofructokinase activity in each group, were measured using the glucose oxidase method. Data are expressed as the mean ± S.D. (*n* = 3/group). Statistical significance: * *p* < 0.05 vs. C, # *p* < 0.05, vs. GP. C: control, GP: high-fat high-glucose (HFHG) group, EX-GP: exendin-4 + HFHG.

We next examined the relevant indicators of glucose metabolism and the TCA cycle and found reduced glucose consumption and lactic acid concentrations, increased pyruvate dehydrogenase E1 (PDH E1) activity, and decreased phosphofructokinase (PFK) activity in the HFHG group. These results suggest that glycolysis and the TCA cycle were weakened. After the pretreatment of mesangial cells with exendin-4, the effects on these metabolic indices were reversed, which suggests that exendin-4 can have a protective role by improving metabolism in cells (Figure 9A–E).

As an important pathway of metabolism, the AMPK signaling pathway has a close relationship with chronic kidney disease and the organ-protective mechanism of exendin-4. We thus pretreated mesangial cells with AICAR, an agonist of AMPK signaling, and found that following this, the survival rate of the cells was increased, the concentration of lactate was decreased, and the activities of PFK, SDH, and PDH-E1 were elevated (*p* < 0.05) compared with those in the high-sugar and high-fat-treated group. These results suggest that the AMPK signaling pathway plays a role in improving the abnormal metabolism induced by HFHG (*p* < 0.05; Figure 9F–J). The aforementioned effects bear resemblance to those observed after the treatment employing exendin-4.

## 4. Discussion

Our results indicate that exenatide significantly reduced UA excretion, suggesting that exenatide might be used to prevent the harmful effects of diabetic nephropathy. Previous studies based on animal models and clinical patients with DKD have revealed that perturbed metabolic pathways, including glycolysis, lipid metabolism, and amino acid metabolism, could be involved in this disease [21]. In this study, we used rat kidney tissue and mesangial cells to determine the crucial metabolic perturbation that primarily contributes to the underlying mechanism of DKD.

Energy production in the kidneys, which are mitochondria-rich organs, occurs largely through respiration. A previous study showed that 12 d of exposure to hyperglycemia significantly diminishes both mitochondrial respiration and glycolytic capacity, suggesting that cells must maintain the balance between the production and consumption of energy [22]. As shown in this study, decreased pyruvate levels and increased lactate levels in the kidneys of diabetic rats could indicate enhanced glycolysis, which can result in the production of more energy for the kidneys. Our study also found significantly low levels of NAD^+^ in the kidneys of diabetic rats, and one study provided evidence that a decrease in NAD^+^ affects the respiratory chain, further leading to a decrease in ATP and thus affecting energy production [23]. To further understand the TCA cycle-related energy metabolites, we tested TCA cycle intermediates.

The TCA cycle plays an important role in renal injury. In the uremic population, a significant relationship between the degree of renal insufficiency and the sum of TCA cycle intermediates in the serum was observed [21]. Other studies also found that TCA cycle-related urinary metabolites are increased in mice with DKD through metabolomic analysis [20]. In our kidney injury model, we found high levels of succinate in kidney tissues, which suggests the accumulation of TCA cycle intermediates. We also found an increased level of glucose, which indicates that less glucose was fed into the TCA cycle or that glucose metabolism via the TCA cycle was less efficient. These results support the hypothesis that there is an insufficient energy supply during diabetic kidney injury, which could be the mechanism that leads to kidney injury.

Renal disease tends to be associated with metabolism through aerobic glycolysis, which, despite being an inefficient method of producing ATP, facilitates the formation of substrates important for the production of biomass via the pentose phosphate pathway [24]. In our study, the levels of some amino acids were perturbed in the DM group, which could be the result of disturbances in amino acid metabolism. Some of the dysregulated amino acids were branched-chain amino acids (BCAAs), including leucine, isoleucine, and valine. Recently, many studies have reported that BCAAs not only play a large role in nutritional regulation, but are also associated with the development of type 2 diabetes, insulin resistance [25], and chronic kidney disease [25]. Mi et al. reported that BCAAs prevent early diabetic kidney injury through a mechanism that might be related to reductions in oxidative stress in the kidneys of rats with STZ-induced diabetes [26]. In our investigation, elevated levels of BCAAs were found in the renal tissues of diabetic rats, possibly indicating a stress response to early kidney injury. Certain amino acids, including alanine, glutamic acid, aspartic acid, glycine, and valine, can be transformed into tricarboxylic acid cycle intermediates, and these include pyruvate, α-ketoglutarate, and oxaloacetate, which take part in the gluconeogenic pathway. This can lead to an increase in these metabolites, possibly influencing the gluconeogenesis process and ultimately exacerbating the disruption of gluconeogenesis [27].

Glycolipid toxicity is thought to be a key factor in the initial adaptation of the heart to the diabetic environment and subsequent maladaptation [28]. Triglycerides are the main lipids synthesized in the liver and kidneys from 3-hydroxybutyric acid (3-HB) [29]. Reusch et al. [30] reported increased levels of 3-HB in diabetic rat kidney tissues, and 3-HB is a component of atherogenic plaques that affects the membrane function. Similarly, in this study, we found elevated levels of 3-HB in the kidney tissues of diabetic rats, which could disturb lipid metabolism and result in kidney injury. PC is an essential component of cell membranes, and disturbed PC metabolism can indirectly affect membrane protein functions. Our study also found decreased levels of PC in the DM group, suggesting kidney injury. TAU plays an important role in the regulation of cellular volume, oxidative status, and Ca^2+^ homeostasis [31]. TAU transporter-knockout mice exhibit significant oxidative damage, endoplasmic reticulum stress, and apoptosis [32]. TAU can also be used to treat kidney infections, kidney stones, and diabetes [33,34]. In this study, low levels of TAU were observed in the DM group, which indicates that the antioxidant capacity was decreased, causing further kidney mitochondrial dysfunction.

In recent years, with increasing knowledge of the renal effects of GLP-1, most research has focused on electrolyte excretion and diuresis [35], tubular reabsorption [36], the glomerular filtration rate [37], and the inhibition of renin–angiotensin system activity [38]. However, the underlying mechanisms remain unclear. Here, we found that exenatide altered kidney metabolism in diabetic rats fed an HFHG diet. Using an NMR-based approach, we observed that exenatide normalized the levels of lactate and glucose. This suggests that exenatide ameliorates energy metabolism and increases the efficiency of the TCA cycle, causing more pyruvate to enter the TCA cycle and less pyruvate to be converted to lactate. This explains why the level of pyruvate change was not statistically significant. Exenatide also increased NAD^+^ and AMP levels, and the resulting energy production was recovered. In addition, changes in the amino acid concentrations in the ex-DM group became properly regulated, indicating that exenatide ameliorated renal injury through amino acid metabolism.

To further clarify the mechanism of energy metabolism, mesangial cells were selected for verification experiments. Renal mesangial cells treated with HFHG showed increased apoptosis, decreased cell survival rate, increased FN expression, reduced Bcl-2/Bax ratio, increased cyt-c and active caspase-3 expression, and weakened mitochondrial antioxidant functions. These factors are closely associated with mitochondrial functions, and these changes were reversed by exendin-4 treatment. The influence of mitochondrial dysfunction on energy metabolism was verified by further assessing energy metabolism indicators. PDH E1 activity was increased and succinate dehydrogenase (SDH) activity was decreased in the GP group, suggesting that the efficiency of the TCA cycle was decreased. In the GP group, decreased PFK activity supported the hypothesis that glycolysis was weak. Exendin-4 treatment rescued abnormal enzyme activity and reduced glucose consumption, suggesting that exendin-4 improves energy metabolism in mesangial cells.

This study inevitably has some limitations. First, kidney cells include podocytes, mesangial cells, and renal tubules. In this study, we were only able to confirm that exendin-4 might improve the function of glomerular mesangial cells through energy metabolism mechanisms, and we did not explore the effects of HFHG and exendin-4 on the two other types of cells. Second, we did not confirm our exendin-4 data using a GLP-1 receptor antagonist. Future studies will include avexitide to further verify the therapeutic effect of exenatide on DKA.

In conclusion, our research showed that exenatide has a protective effect against HFHG-mediated diabetic kidney injury in vivo and in vitro. Exenatide improves energy metabolism by ameliorating the abnormal metabolism of amino acids, lipids, and the TCA cycle. In glomerular mesangial cells, exendin-4 improves energy metabolism by enhancing mitochondrial function, thereby inhibiting HFHG-mediated cytotoxicity. Therefore, exenatide treatment might be an effective therapy for diabetic kidney injury, and metabolic studies could be a possible approach to identifying novel therapies for this condition.

## Figures and Tables

**Table 1:** Effects of exenatide on kidney functions in HFHS-treated rats.

	NC	DM	Ex-DM
Renal index	3.34 ± 0.4	6.63 ± 0.7 *	4.69 ± 0.9 #
BUN (mmol/L)	6.6 ± 1.4	7.7 ± 2.1	7.1 ± 2.9
CREA (µmol/L)	50.36 ± 9.0	61.25 ± 8.5	51.23 ± 4.1
24 h UMA (mg)	69.7 ± 8.5	494.7.2 ± 101.6 *	425.3 + 95.2 #

Data are expressed as the mean ± S.D. (*n* = 6/group). Statistical significance: * *p* < 0.05 vs. NC, # *p* < 0.05 vs. DM. NC: control; DM: diabetes mellitus (DM)-model rat group; Ex-DM: exenatide-treated DM-model rat group; BUN, blood urea nitrogen; CREA, creatinine.

**Table 2:** Identified metabolites in ^1^H NMR spectra of kidney tissue aqueous extracts.

NO.	Metabolites	δ ^1^H (ppm) and Multiplicity	Moieties
1	Leucine	0.95 (d), 0.96 (d), 1.70 (m), 1.70 (m), 1.73 (m), 3.73 (m)	α-CH_3_, α-CH_3_, γ-CH, β-CH_2_, α-CH
2	Isoleucine	0.92 (t), 1.00 (d), 1.24 (m), 1.45 (m), 1.97 (m), 3.66 (d)	δ-CH_3_, γ-CH_3_, half γ-CH_2_, half γ-CH_2_, β-CH, α-CH
3	Valine	0.98 (d), 1.03 (d), 2.26 (m), 3.61 (d)	γ-CH_3_, γ-CH_3_, β-CH, α-CH
4	Alanine	1.48 (d), 3.77 (dd)	β-CH_3_, α-CH
5	Acetate	1.92 (s)	CH_3_
6	Glutamate	2.04 (m), 2.12 (m), 2.33 (m), 2.37 (m), 3.75 (dd)	half β-CH_2_, half β-CH_2_, half γ-CH_2_, half γ-CH_2_, α-CH
7	Aspartate	2.68 (dd), 2.80 (dd), 3.89 (dd)	β-CH_2_; α-CH
8	Creatine	3.02 (s), 3.92 (s)	N-CH_3_, α-CH_2_
9	Glycine	3.57 (s)	α-CH_2_
10	Glucose	β (3.24 (dd), 3.48 (t), 3.90 (dd)), α (3.54 (dd), 3.71 (t), 3.72 (dd), 3.83 (m))	β(H_2_, H_3_, H_5_), α(H_2_, H_3_, H_6_)
11	Myo-inositol	3.28 (t), 3.53 (dd), 3.63 (t), 4.07 (t)	^2^CH, ^4,6^CH, ^1,3^CH, ^5^CH
12	Lactate	1.32 (d), 4.10 (q)	β-CH_3_, α-CH
13	Fumarate	6.51 (s)	CH
14	Tyrosine	3.05 (dd), 3.19 (dd), 6.90 (d), 7.19 (d)	half β-CH_2_, half β-CH_2_, β-CH, α-CH
15	Phenylalanine	3.12 (dd), 3.30 (dd), 3.99 (dd), 7.32 (d), 7.37 (t), 7.42 (t)	α-CH, half β-CH_2_, half β-CH_2_, α-CH, β-CH, γ-CH
16	NAD^+^	6.03 (d), 6.08 (s), 8.18 (s), 8.21 (m), 8.43 (s), 8.82 (d), 9.15 (d), 9.34 (s)	NH_2_, NH_2_(CO), δ-CH, β-CH, ^2^CH, γ-CH, α-CH
17	AMP	6.14 (d), 8.25 (s), 8.57 (s)	NH_2_, δ-CH, 2CH
18	Methionine	2.01 (m), 2.14 (s), 2.16 (m), 2.64 (t), 3.86 (m)	δ-CH_3_, γ-CH_2_, β-CH_2_
19	Pyruvate	2.37 (s)	CH_3_
20	Succinate	2.41 (s)	2CH
21	Glycerol	3.57 (dd), 3.62 (dd), 3.79 (m)	half ^1^CH_2_, half ^3^CH_2_, ^2^CH
22	Lysine	1.43 (m), 1.49 (m), 1.70 (m), 1.90 (m), 3.02 (t), 3.75 (t)	half γ-CH_2_, half γ-CH_2_, δ-CH_2_, β-CH_2_, ε-CH_2_, α-CH
23	Citrate	2.54 (d), 2.69 (d)	half-CH_2_, half-CH_2_
24	3-Hydroxybutyrate	1.19 (d), 2.30 (dd), 2.39 (dd), 4.14 (m)	CH_3_, half α-CH_2_, half α-CH_2_, γ-CH
25	Betaine	3.31 (s), 3.91 (s)	3CH_3_, α-CH_2_
26	Hypoxanthine	8.19 (s), 8.21 (s)	CH, CH
27	Inosine	3.79 (dd), 3.89 (dd), 4.28 (dd), 4.44 (dd), 4.78 (t), 6.09 (d), 8.22 (s), 8.34 (s)	half-CH_2_, half-CH_2_, CH, CH, CH, CH, CH
28	Taurine	3.27 (t), 3.43 (t)	^1^CH_2_, ^2^CH_2_
29	Phosphocholine	3.22 (s), 3.60 (t), 4.18 (m)	N-(CH_3_)_3_, N-CH_2_, CH_2_OH
30	GPC	3.23 (s), 3.60 (dd), 3.68 (dd), 3.87 (m), 3.94 (m), 4.33 (m)	N-(CH_3_)_3_, half ^1^CH_2_, ^2^CH_2_, half ^1^CH_2_, half ^3^CH_2_, half ^3^CH_2_, ^1^CH_2_
31	Choline	3.21 (s), 3.51 (dd), 4.04 (t)	N-(CH_3_)_3_, N-CH_2_, CH_2_OH
32	Uracil	7.56 (d), 5.81 (d)	α-CH, β-CH
33	Niacinamide	8.9 (dd), 8.7 (dd), 8.2 (m), 7.6 (m)	N(CH), δ-CH, β-CH, γ-CH

## Data Availability

The data presented in this study are available on request from the corresponding author. The data are not publicly available due to confidentiality.

## Abbreviations

Diabetic kidney disease (DKD), diabetes mellitus (DM), high-fat high-glucose (HFHG), adenosine 5′-monophosphate (AMP)-activated protein kinase (AMPK), glucagon-like peptide-1 (GLP-1), urinary albumin (UA), high-density lipoprotein (HDL), low-density lipoprotein (LDL), blood urea nitrogen (BUN), creatinine (CREA), fibronectin (FN), glutathione reductase (GR), branched-chain amino acids (BCAAs), succinate dehydrogenase (SDH), phosphofructokinase (PFK), pyruvate dehydrogenase activity E1 (PDH E1), nicotinamide adenine dinucleotide (NAD^+^), adenosine monophosphate (AMP), taurine (TAU), phosphocholine (PC).
